# Assessment of (‐) epicatechin as natural additive for improving safety and functionality in fresh “Piel de Sapo” melon juice

**DOI:** 10.1002/fsn3.2251

**Published:** 2021-03-30

**Authors:** Javier Rúa Aller, Sonia González González, Javier Sanz Gómez, Maria Pilar del Valle Fernandez, María Rosario García‐Armesto

**Affiliations:** ^1^ Department of Molecular Biology University of León León Spain; ^2^ Food Science and Food Technology Institute León Spain; ^3^ Department of Food Hygiene and Food Technology University of León León Spain

**Keywords:** “Piel de sapo” melon juice, epicatechin, pathogenic bacteria, probiotic type‐lactic acid bacteria

## Abstract

Epicatechin (EC) is a very abundant flavonoid in vegetable tissues that presents high antioxidant activity in living systems. The minimum inhibitory concentration (MIC) of (‐)EC was determined in three species of bacteria commonly associated with foodborne illness of plant origin: *Listeria* (*L*.) *monocytogenes*, *Escherichia* (*E*.) *coli* ‐serogroups O157: H7 and O111‐ and *Bacillus* (*B*.) *cereus*; two strains of probiotic‐type lactic acid bacteria (PT‐LAB) and two control strains. All 10 strains were assayed under three temperature conditions (30º, 10º, and 4ºC) and at each temperature under two pH conditions (6.7 and 5.5). Mean EC MIC values were generally lower at refrigeration (4º and 10ºC) temperatures and at standard pH (6.7). By inoculating with each of the strains separately, both melon juice (MJ) and MJ supplemented with EC (ECSMJ), at the accepted maximum sensorial limit, and storing them at 4ºC for 10 days; the final counts (CFU/mL) were lower for ECSMJ than for plain MJ both for pathogenic bacteria and for PT‐LAB. The presence of EC during refrigerated storage counteracted the ability of MJ as a growth medium for all the pathogenic bacteria. ECSMJ increased the antioxidant activity of MJ significantly to levels similar to those of EC alone. (‐) Epicathechin would be a promising ingredient for increasing the functional properties of “Piel de Sapo” MJ (phenolic compounds and antioxidant ability) while contributing to improving the safety of this type of juice during prolonged refrigerated storage at 4ºC.

## Graphical abstract

- (‐) Epicatechin is a very abundant flavonoid in vegetable tissues that presents high antioxidant activity in living systems.
- (‐) Epicathechin would be a promising ingredient for increasing the functional properties of “Piel de Sapo” MJ (phenolic compounds and antioxidant ability) whilst contributing to improving the safety of this type of juice during prolonged refrigerated storage at 4 ºC.

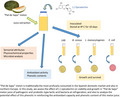

## 1 INTRODUCTION

“Piel de Sapo” is a variety of melons belonging to *Cucumis melo* L. var. *saccharinus* (*Inodorus* varietal group).(Condés & Hoyos, 2008) It is traditionally the most profusely consumed in the Spanish domestic market and also, nowadays, increasingly sold in northern Europe.(CBI‐NL,) This group of melons is characterized by its low‐calorie content, refreshing properties, and pleasant sweet taste. However, the *Inodorus* varietal group differs from other common varietal groups of melons in the market (*Cantaloupensis*) in that it has fewer biologically active compounds, such as vitamin A, β‐carotene, vitamin C, and total phenols.( Amaro et al., 2015).

Foodborne illness attributed to melons has become a significant public health concern in some countries in recent decades.(USDA‐FDA, 2018; Walsh et al., 2014) The most common etiological agents involved are *Salmonella enterica* (particularly associated with the netted *Cantaloupensis* varietal group), although other bacterial agents have also been reported, among which are verotoxin‐producing *E. coli* – VTEC – (mostly serotypes O157:H7 and O111), *L. monocytogenes,* and *B. cereus*.(Salomão et al., 2018; Walsh et al., 2014).

In order to increase the safety of fresh juices, the addition of natural antimicrobials found in edible plants and herbal extracts has recently been proposed among other alternatives.(Rudra et al., 2020) E**picatechin** (EC) is a very abundant flavonoid in vegetable tissues that presents high antioxidant activity in living systems. Specifically, it is included in the flavanols group, one of the five major polyphenolic groups found in tea leaves and various apple varieties.(Mendoza‐Wilson & Glossman‐Mitnik, 2006; Tsao et al., 2003) Flavanols exhibit the highest radical scavenging activity (4.21 mM), significantly more than other flavonoids. However, little is known about the anti‐microbial effect of EC on foodborne pathogens, although it has been reported that EC displays anti‐bacterial activity against *Helicobacter pylori*.( Escandón et al., 2016) Also, to our knowledge, no information exists on the effect of EC in PT‐LAB, although polyphenols have recently been recognized as a candidate category of prebiotic compounds.(Gibson et al., 2017).

Previous studies in our lab have indicated that “Piel de Sapo” melon juice (MJ) enables the viability of verotoxin‐producing *E. coli* (VTEC) and growth potential (δ) of PT‐LAB at 4ºC for 4 days. (Rúa et al., 2018, 2019) The aim of the present research was to assess the effect of a natural phytochemical EC on viability and growth in “Piel de Sapo” MJ of pathogenic (VTEC – O157:H7 and O111‐, *L. monocytogenes* and *B. cere*us) and of PT‐LAB (*Lactobacillus* [*Lb*.] *rham*nosus GG and *Lactococcus* [*Lc*.] *lactis* subsp. *cremoris* 660) bacteria at 4ºC for 10 days and also to analyze the potential effect of EC in reinforcing the antioxidant capacity and phenolic content of this MJ.

## 2 MATERIALS AND METHODS

### 2.1 Bacterial strains and growth conditions

The strains used to carry out the study belong to three groups: (A) PT‐LAB: *Lactobacillus* (*Lb*.) *rhamnosus*, ATCC 53,103, and *Lactococcus* (*Lc*.) *lactis*, strain 660; (B) strains commonly associated with foodborne illnesses in vegetable products: *Escherichia* (*E*.) *coli* O157:H7, ATCC 700,728; *E. coli* O111, strain 172; *Listeria* (*L*.) *monocytogenes* CECT 4,032, serovar 4b; *L. monocytogenes,* strain L 74, *Bacillus* (*B*.) *cereus*, strain GTE 216 and *B. cereus*, strain HRM‐1; (C) control strains: *Enterococcus* (*En*.) *faecalis*, ATCC 29,212 and *E. coli,* ATCC 25,922. Additional information on the bacterial strains are shown in (Table 1; Caro, 2004; García‐Armesto et al., 1993; García‐Armesto & Sutherland, 1997; Monteagudo‐Mera et al., 2011, 2012).

**TABLE 1:** Bacterial strains used in this study, their sources, and other characteristics

Genera/species	Strain	Origin/isolation	Comments	Application
*Lactobacillus rhamnosus*	ATCC 53,103	Human feces	Other designation: strain GG (Gorbach‐Goldin)	Produces antibacterial agent. Inhibits colon disorders. PT‐LAB( Monteagudo‐Mera et al., 2011, 2012)
*Lactococcus lactis* subsp. *cremoris*	660^1^	Raw ewes` milk	Psychrotrophic( García‐Armesto et al., 1993)	Produces antibacterial agent PT‐LAB ( Monteagudo‐Mera et al., 2011, 2012)
*Enterococcus faecalis*	ATCC 29212^1^	Urine	Verified by whole‐genome sequencing	Susceptibility testing, CLSI quality control strain.
*Bacillus cereus*	GTE 216	Milk	Psychrotrophic( Caro, 2004; García‐Armesto & Sutherland, 1997)	
*Bacillus cereus*	HRM−1^1^	Milk	Psychrotrophic( Caro, 2004; García‐Armesto & Sutherland, 1997)	
*Listeria monocytogenes*	CECT 4,032 NCTC 11,994	Isolated by Pini, P.*N*.	Serovar 4b, β‐haemolysis (sheep) Associated with case of meningitis after eating soft cheese	Reference strain recommended to be used for UNE‐CEN ISO/TS 11,133
*Listeria monocytogenes*	L74^1^	Spanish sausage		
*Escherichia coli* O157:H7	ATCC 700,728	*N*.S.	Absence of verotoxin genes Confirmed by PCR	Quality control strain for BBL Chromagar
*Escherichia coli* O111	172^1^	Ewes` milk	*stx_1_^‐^, stx_2_^‐,^*	
*Escherichia coli* O6	ATCC 25,922	Clinical	Biotype 1 Does not produce verotoxin	CLSI control strain for antimicrobial susceptibility testing

Note

Abbreviation: ATCC, American Type Culture Collection; CECT, Colección Española de Cultivos Tipo; NCTC, National Collection of Type Cultures; *N*.S, non specified.

^1^ Isolates from Collection of Department of Food Hygiene and Food Technology, University of León (Spain).

The test strains were kept cryopreserved in 50% (v v^‐1^) glycerol. Frozen stock cultures were activated by transferring them to Tryptone Soya Broth (TSB) + 0.6% (w v^‐1^) yeast extract (TSBYE) broth and incubating at 35°C for 24 hr. After incubation, they were plated to check the purity of the strains: *E. coli* in Tryptone Bile Agar with X‐Glucuronide, Biokar (TBX) at 44°C for 24 hr and the rest of the bacteria in Tryptone Soya Agar, Biokar (TSA) at 30 ºC for 24 hr. The isolated pure colonies were inoculated in TSBYE broth and incubated at the optimum temperatures for each group of strains, obtaining a pure culture for experimental studies.

### 2.2 Chemicals and preparation of (‐)‐epicatechin stock solution

ABTS (2,2’‐azino‐bis(3‐ethylbenzothiazoline‐6‐sulfonic acid), DPPH (2,2‐diphenyl‐1‐picryl‐hydrazyl), (‐) EC, Fast Blue BB (4‐benzoylamino‐2,5‐dimethoxybenzenediazonium chloride hemi‐[zinc chloride] salt), ferric chloride (FeCl_3_), gallic acid, Folin & Ciocalteau's Phenol Reagent, potassium persulfate, TPTZ (2,4,6, trypyridyl‐S‐triazine), and Trolox (6‐hydroxy‐2,5,7,8 tetramethylchroman‐2‐carboxylic acid) were obtained from Sigma‐Aldrich Co. (St. Louis, MO, USA).

The EC stock solution (10 mg/ml in 40% ethanol) was stored frozen. Before working with this solution, the sensitivity of the strains to ethanol in microtiter plate was checked by serial dilutions, starting from 40% ethanol up to 0.65% (v v^‐1^); thus was found that the antimicrobial effect was found to be due to EC only.

### 2.3 MIC and MBC assays

The antimicrobial microdilution assay to estimate the minimum inhibitory concentration (MIC) values of EC against all the strains was carried out according to ISO 10,932:2010.( ISO, 2010) Cation adjusted Mueller Hinton Broth (CA‐MHB) was used in the assay at pH 6.7 (standard) or 5.5 (resembling that of MJ) by adjusting the broth with 1 mol/L HCl. The experiments were carried out at 30ºC (which allows the growth of all strains) for 1 day, 10ºC (abuse temperature) for 7 days, and 4ºC (recommended refrigeration temperature for the fresh MJ) for 10 days. The MIC value was considered the minimal concentration of antimicrobial compound that inhibits visible growth of the strain tested.(Barry, 1976) At least three independent tests were performed in duplicate with each strain. Minimal bactericidal concentration (MBC) was estimated from the same microplates used to determine the MIC and it was defined as the lowest concentration of antimicrobial compound resulting in a 99.9% kill of the viable cell in the primary inoculum.(Barry, 1976).

### 2.4 Preparation and characterization of MJ

“Piel de Sapo” MJ was prepared and characterized as previously reported.(Rúa et al., 2018) Samples of MJ, both plain (MJ) and supplemented with 5,000, 2,500 or 1,250 µg/ml EC (ECSMJ), were subjected to sensory evaluation at the beginning (time 0) of the experiment and after 10‐day storage at 4ºC by six trained panelists from the Institute of Food Science and Technology of the University of León. Also physical‐chemical analyses (pH, titratable acidity and ºBrix) was performed on MJ and ECSMJ at time 0 and after 10‐day storage at 4ºC.(Rúa et al., 2018).

### 2.5 Antioxidant activity and total phenolic contents

Antioxidant activity was determined by the ABTS, DPPH, and FRAP methods and total phenolic contents (TPC) was determined using the Folin‐Ciocalteau method (FC) and the Fast Blue BB (FBBB) method. Results for antioxidant activitiy were expressed as µmol Trolox per 100 ml of sample, and the results for TPC were expressed as mg of gallic acid equivalents per 100 ml. These methods were described previously (Rúa et al., 2018) Stock samples of MJ, EC, and ECSMJ (2,500 µg/ml of EC in the juice) were stored frozen. Duplicate aliquots of these samples were thawed, kept for 0 (2 hr), 1, 3, 5, 7, and 10 days at 4ºC and analyzed in duplicate for their antioxidant activity and TPC.

### 2.6 Survival and growth of inoculated bacteria in melon juice

Freshly squeezed MJ and ECSMJ at a final EC concentration of 2,500 µg/ml were inoculated separately with 10^5^ CFU/mL of one of PT‐LAB and 10^3^– 10^4^ CFU/mL for the rest of the bacteria strains used in this study. Survival and growth of the strains were assessed at 0, 1, 3, 5, 7, and 10 days at 4ºC by counting on TBX for *E. coli* strains, Plate Count Agar (PCA) for *B. cereus* and *En. faecalis* strains, and TSA for *L. monocytogenes* and PT‐LAB strains. The experiments were done in two batches of MJ, each under two conditions (MJ and ECSMJ). The growth potentials (δ) for each condition and each of the 10 strains were calculated as the difference between growth after 10‐day storage in comparison with that at the beginning of the experiment (*t* = 0). The highest growth potential value obtained for the two lots in duplicate was considered. The results were interpreted according to criteria used by Beaufort *et al*.Beaufort et al., 2014), considering that a δ > 0.5 log_10_ indicates that MJ and ECSMJ are able to support the growth of the bacteria tested.

### 2.7 Statistical analysis

Statistical analysis was carried out using a one‐way analysis of variance (ANOVA) for comparison of more than two different groups, using the posthoc Tuckey and Duncan method. The ANOVA analysis was performed using the statistical analysis program SPSS Statistics version 24 for Windows, available on the IBM website.

## 3 RESULTS AND DISCUSSION

### 3.1 Antimicrobial activity of EC in growth media under different temperature and pH conditions

Two PT‐LAB, six strains with potential pathogenic ability, and two control bacteria were used in this study (Table 1). Under the tested conditions of pH and temperature, in the culture media used (CA‐MHB), significant differences were found in the antimicrobial activity (MIC values) of EC (Table 2). The highest MIC values were observed for *B. cereus* HRM‐1 under all conditions (5,011.50 µg/ml) and the lowest for *E. coli* ATCC 25,922 (234.91 µg/ml at 10ºC, pH 6.7). In general (mean MIC values for the 10 bacteria), EC MICs were lower at refrigeration temperatures (10 and 4ºC) than at 30ºC, regardless of the assay culture broth pH (standard or 5.5). Therefore, these results indicate that refrigeration temperatures have a specific influence on the MIC values of EC. The MIC values were higher at the pH resembling that of the MJ (5.5) than at the pH of the standard broth assay (6.7) at 30ºC (1.5 times) and 10ºC (2.4 times); however, MICs were slightly lower at 4ºC.

**TABLE 2:** Antibacterial activity (MIC) of (‐) epicatechin (µg/mL) against the strains used in this study under the conditions indicated

Bacteria tested	Minimum inhibitory concentration (MIC) (µg/mL)					
	30 ºC ^1^/ pH 6.7	30 ºC / pH 5.5	10 ºC^2^ /pH 6.7	10 ºC / pH 5.5	4 ºC^3^ / pH 6.7	4 ºC / pH 5.5
*Lb. rhamnosus* ATCC 53,103	2,216.63 ± 1,368.05^ab^	3,758.62 ± 1,339.38^a^	1,252.88 ± 0.00^bc^	5,011.50 ± 0.00^c^	1,252.88 ± 0.00^b^	1722.70 ± 648.42^c^
*Lc. lactis* 660	2,863.71 ± 1508.94^b^	3,758.62 ± 1,339.38^a^	626.44 ± 0.00^ab^	1879.32 ± 686.22^ab^	469.83 ± 165.08^a^	814.37 ± 302.60^a^
*En. faecalis* ATCC 29,212	1989.86 ± 635.58^ab^	4,100.32 ± 1,264.21^a^	626.44 ± 0.00^ab^	1,252.88 ± 0.00^a^	495.93 ± 207.17^a^	1,357.28 ± 615.90^bc^
*B. cereus* GTE	2,505.75 ± 0.00^ab^	4,295.57 ± 1,222.68^a^	1,610.84 ± 611.34^c^	5,011.50 ± 0.00^c^	5,011.50 ± 0.00^d^	5,011.50 ± 0.00^e^
*B. cereus* HRM−1	5,011.50 ± 0.00^c^	5,011.50 ± 0.00^a^	5,011.50 ± 0.00^d^	5,011.50 ± 0.00^c^	5,011.50 ± 0.00^d^	5,011.50 ± 0.00^e^
*L. monocytogenes* CECT 4,032	2,255.18 ± 528.25^ab^	3,758.62 ± 1,339.38^a^	1,017.96 ± 324.21^bc^	5,011.50 ± 0.00^c^	5,011.50 ± 0.00^d^	1,252.88 ± 0.00^b^
*L. monocytogenes* L 74	1,252.88 ± 0.00^a^	3,341.00 ± 1,293.96^a^	939.66 ± 343.11^b^	3,132.19 ± 1534.45^b^	1,258.88 ± 0.00^b^	2,505.74 ± 0.00^d^
*E. coli* ATCC 700,728	2,505.75 ± 0.00^ab^	4,009.20 ± 1,293.96^a^	1,252.88 ± 0.00^bc^	1,252.88 ± 0.00^a^	1,044.07 ± 323.49^b^	626.44 ± 0.00^a^
*E. coli* 172	2,505.75 ± 0.00^ab^	3,758.63 ± 1,339.38^a^	1,252.88 ± 0.00^bc^	1809.71 ± 854.60^ab^	2,662.36 ± 1,045.55^c^	1,252.88 ± 0.00^b^
*E. coli* ATCC 25,922	2,326.77 ± 1,286.83^ab^	3,132.19 ± 1696.40^a^	234.91 ± 85.77^a^	1,252.88 ± 0.00^a^	313.22 ± 0.00^a^	469.83 ± 171.56^a^

Note

All values are Mean ± Standard Deviation. Mean values in the same column following by different letters are significantly different (*p* <.05).

^1^ 1 day of culture.

^2^ 7 days of culture.

^3^ 10 days of culture.

Mean MBC values for each temperature and pH value were higher than the MIC values corresponding to the same conditions, with five exceptions (Table 3). MBCs were also generally higher at pH 5.5 than at pH 6.7, regardless of the temperature used. At pH 6.7, there was more diversity in the behaviour of the strains (two groups at 10 and 4ºC; and three at 30ºC) compared to that obtained at pH 5.5 (one group at 30 and 4ºC and two groups at 10ºC). Taking into account that an antimicrobial compound is considered bactericidal when the MBC has a value less than double that of its MIC, and bacteriostatic when it is higher than double, (Moody et al., 2007) the effect for EC would be bactericidal for all strains at 30ºC and the two pH values and mostly bacteriostatic at 10 and 4ºC for the two pHs.

**TABLE 3:** Antibacterial activity (MBC) of (‐) epicatechin (µg/mL) against the strains used in this study under the conditions indicated

Bacteria tested	Minimum bactericidal concentration (MBC) (µg/mL)					
	30 ºC ^1^/ pH 6.7	30 ºC / pH 5.5	10 ºC^2^ /pH 6.7	10 ºC/pH 5.5	4 ºC^3^ / pH 6.7	4 ºC / pH 5.5
*Lb. rhamnosus* ATCC 53,103	3,340.99 ± 1,293.97^ab^	5,011.50 ± 0.00^a^	2,505.75 ± 1,640.39^ab^	5,011.50 ± 0.00^b^	3,758.62 ± 1,339.38^ab^	5,011.50 ± 0.00^a^
*Lc. lactis* 660	3,758.62 ± 1,339.38^b^	5,011.50 ± 0.00^a^	2,505.75 ± 0.00^ab^	3,341.00 ± 1,293.96^a^	5,011.50 ± 0.00^b^	5,011.50 ± 0.00^a^
*En. faecalis* ATCC 29,212	2088.12 ± 626.43^a^	5,011.50 ± 0.00^a^	2,505.75 ± 0.00^ab^	3,341.00 ± 1,293.96^a^	>5,011.50	>5,011.50
*B. cereus* GTE	3,221.67 ± 1,222.68^ab^	4,295.57 ± 1,222.68^a^	1879.32 ± 723.34^a^	>5,011.50	>5,011.50	>5,011.50
*B. cereus* HRM−1	5,011.50 ± 0.00^c^	>5,011.50	>5,011.50	>5,011.50	>5,011.50	>5,011.50
*L. monocytogenes* CECT 4,032	2,505.75 ± 0.00^ab^	4,295.57 ± 1,222.68^a^	3,579.64 ± 1,339.38^b^	>5,011.50	>5,011.50	>5,011.50
*L. monocytogenes* L 74	2,505.75 ± 0.00^ab^	4,593.87 ± 1,022.97^a^	2,505.75 ± 0.00^ab^	5,011.50 ± 0.00^b^	>5,011.50	>5,011.50
*E. coli* ATCC 700,728	2,505.75 ± 0.00^ab^	5,011.50 ± 0.00^a^	2,505.75 ± 0.00^ab^	5,011.50 ± 0.00^b^	2,505.75 ± 0.00^a^	4,176.25 ± 1,293.96^a^
*E. coli* 172	2,505.75 ± 0.00^ab^	5,011.50 ± 0.00^a^	2,505.75 ± 0.00^ab^	3,758.63 ± 1940.94^ab^	3,758.62 ± 1,339.38^ab^	4,176.25 ± 1,293.96^a^
*E. coli* ATCC 25,922	2,505.75 ± 0.00^ab^	5,011.50 ± 0.00^a^	1754.03 ± 646.98^a^	4,176.25 ± 1,293.96^ab^	2,505.75 ± 0.00^a^	4,176.25 ± 1,293.96^a^

Note

All values are Mean ± Standard Deviation. Mean values in the same column following by different letters are significantly different (*p* <.05).

^1^ 1 day of culture.

^2^ 7 days of culture.

^3^ 10 days of culture.

Little is known about the antimicrobial effect of EC, however there are several studies with other catechins, mainly (‐)‐epigallocatechin (EGC) and (‐)‐epigallocatechin gallate (EGCG), of green tea against the growth of Gram‐negative and Gram‐positive bacteria. With regard to the two PT‐LAB assayed, strain *Lb. rhamnosus* ATCC 53,103 was, in general, more resistant to EC than *Lc. lactis* 660. Similarly, Lee *et al* Lee et al., 2006), found that *Lb. rhamnosus* GG was more resistant (<10% inhibition) to the action of phenolic compounds including EC. In addition, MIC values for EC were lower at 4ºC than at 30ºC and at pH 6.7 with respect to pH 5.5; this effect was observed in a previous study(Rúa et al., 2018), but using PLX^®^ as antimicrobial against these PT‐LAB.

The strains of *L. monocytogenes* used in this study are of different origin (cheese and sausage), and this could explain the differences in MICs obtained in some of the studied conditions. In general, *L. monocytogenes* CECT 4,032 is more resistant to EC than the L74 strain. Bubonja‐Sonje *et al*.(Bubonja‐Sonje et al., 2011) reported a MIC value of 3,733 µg/ml for *L. monocytogenes* strain EGD at 4ºC for EC extracted from olive oil, which is in the range of our MICs at this temperature.

The MIC values of EC for the three strains of *E. coli* used in this study ranged from approximately 300 to 4,000 μg/mL. A MIC value > 1,145 μg/mL was described for *E. coli* K12 strain C6.(Ikigai et al., 1993) The use of green tea extract (≤ 4,000 µg/ml) inhibits the growth of *E. coli* fron urinary tract.(Reygaert & Jusufi, 2013).

It has been described that the mode of antibacterial action of green tea extract, EGCG, and EC caused damage to the membranes.(Ikigai et al., 1993) Also, the stability of tea catechins is pH‐ and temperature‐dependent. Tea catechins in aqueous solutions are very stable when pH is below 4; whereas, they are unstable in solutions with pH > 6.0(Ananingsih et al., 2013), which could partly explain the lower antimicrobial effect of EC (MICs) under the conditions used in our study.

### 3.2 Sensorial and physicochemical analysis of MJ and ECSMJ

Two batches of fresh “Piel de Sapo” MJ were sensory evaluated, without supplementation and supplemented with different EC concentrations (5,000, 2,500, and 1,250 µg/ml) (ECSMJ) in order to determine the concentration that the consumer allow. According to the tasting panel, 2,500 μg/mL of EC did not generally modify many of the sensorial parameters tested at the onset of addition and at the end of storage (10 days at 4°C) for fresh MJ or ECSMJ (Table 4). Only a variation in one aspect (fiery taste) of the trigeminal sensation was observed in ECSMJ: the feeling of overheating in the oral cavity (such as that produced by alcohol, pepper, and chilli); this sensation was maintained in the sample stored at 4ºC for 10 days. Possibly, the higher amount of solids and suspended material in the samples affected general acceptability of MJ by the panelist with a mean score of 3.33, indicating liked slightly to disliked moderately. Furthermore, the general acceptability in ECSMJ is lower (3.00), due to the fiery taste caused by the addition of EC. However, further studies would be interesting to perform in order to complete the sensory evaluation of ECSMJ using other consumer acceptability tests, such as the 9‐point hedonic scale (which are the most used) and the “chech‐all‐that‐apply” (CATA) questions.

**TABLE 4:** Sensory scores of “Piel de Sapo” melon juice plain and supplemented with EC (2,500 µg/ml) at the onset of the addition and after 10 days of storage at 4 ºC

Parameter name	MJ		ECSMJ	
	0	10 days	0	10 days
Appearance	4.33	4.33	4.33	4.33
Color	4.16	4.16	4.16	4.16
Flavor	4.16	4.16	4.16	4.16
Melon taste	4.33	4.33	4.33	4.33
Cucumber taste	3.00	3.00	3.00	3.00
Fiery	0	0	3.66	3.66
General acceptability	3.33	3.33	3.00	3.00

Note

1 = extremely dislike, 2 = dislike, 3 = neither like nor dislike, 4 = like; 5 = extremely like. Values are means of two independent determinations by duplicate.

Abbreviations: ECSMJ, plain melon supplemented with EC; MJ, plain melon juice.

With respect to the evolution of the physicochemical parameters at the onset and end of the refrigeration period (10 days), the addition of EC to MJ, barely produced changes in total sugars (ºBrix), pH or acidity (% citric acid) in comparison with untreated juice, with mean values of 5.87 ± 0.20 pH, 10.97 ± 0.78 ºBrix, and 0.034 ± 0.005% citric acid. These values are similar to those we have previously reported.(Rúa et al., 2018) Also, few or no variations in these physicochemical parameters have been reported in some fruit juices subjected to non‐thermal technology.(Tomadoni et al., 2017).

### 3.3 Antioxidant activity and phenolic content of ECSMJ

The antioxidant activity was determined in MJ, a solution of EC (2,500 µg/mL) and in ECSMJ for 10 days stored at 4°C, using three methods: ABTS, DPPH, and FRAP (Table 5). The values for antioxidant activity in MJ were similar using the three methods. The addition of EC to MJ produced a significant increase in the antioxidant activity of ECSMJ, with values similar to those for EC, which was ten times more than that produced by PLX^®^ in MJ, according to a previous study.(Rúa et al., 2018).

**TABLE 5:** Antioxidant activity and total phenolics of epicatechin, “Piel de Sapo” plain melon juice and supplemented with (‐) epicatechin (2,500 µg/ml), stored for 10 days at 4 ºC (mean ± standard deviation)

Methods	Time (days)	E	MJ	ECSMJ
ABTS^1^	0	4,471 ± 307^a^	33.85 ± 4.29^a^	3,814 ± 186^a^
	1	4,259 ± 357^a^	36.67 ± 2.52^a^	3,408 ± 287^ab^
	3	4,107 ± 367^ab^	31.54 ± 1.47^a^	3,718 ± 277^ab^
	5	4,060 ± 116^ab^	21.67 ± 2.08^b^	3,367 ± 396^ab^
	7	4,174 ± 196^a^	32.00 ± 3.91^a^	3,906 ± 313^a^
	10	3,552 ± 247^b^	21.16 ± 1.85^b^	3,260 ± 137^b^
DPPH^1^	0	2,977 ± 270^a^	33.30 ± 2.36^ac^	2,871 ± 234^a^
	1	2,266 ± 490^b^	33.89 ± 2.76^ac^	3,344 ± 191^bc^
	3	3,028 ± 181^a^	37.88 ± 1.64^a^	3,518 ± 303^b^
	5	3,053 ± 406^a^	30.40 ± 2.73^bc^	3,042 ± 234^ac^
	7	2,907 ± 245^a^	25.90 ± 1.27^b^	3,033 ± 262^ac^
	10	2,660 ± 83^ab^	29.20 ± 2.06^bc^	2,887 ± 208^a^
FRAP^1^	0	1,307 ± 143^ab^	45.33 ± 3.72^ab^	1,418 ± 129^a^
	1	1,454 ± 137^a^	48.88 ± 1.67^bc^	1,356 ± 11^a^
	3	1,143 ± 114^b^	42.94 ± 1.26^a^	1,091 ± 35^b^
	5	1,281 ± 159^b^	44.50 ± 1.73^ab^	1,276 ± 108^ac^
	7	1,225 ± 44^b^	43.25 ± 3.30^a^	1,144 ± 55^bc^
	10	1,284 ± 87^ab^	53.50 ± 1.91^c^	1,281 ± 59^ac^
FBBB^2^	0		5.93 ± 0.67^a^	2,163 ± 137^a^ (273.85 ± 17.40)
	1		4.68 ± 0.09^b^	2,112 ± 126^a^ (267.34 ± 15.94)
	3		7.03 ± 0.49^a^	2,288 ± 11^a^ (289.65 ± 1,36)
	5		6.18 ± 0.35^a^	2,300 ± 180^a^ (277.20 ± 8.88)
	7		6.43 ± 0.18^a^	2,112 ± 59^a^ (267.36 ± 7.50)
	10		6.04 ± 0.60^a^	2,128 ± 68^a^ (269.32 ± 8.66)
FC^2^	0		12.88 ± 1.65^a^	104.71 ± 10.52^a^
	1		13.17 ± 1.33^a^	145.35 ± 19.36^b^
	3		19.12 ± 1.75^b^	128.08 ± 19.44^ab^
	5		10.90 ± 0.76^c^	124.27 ± 23.67^ab^
	7		12.94 ± 1.21^a^	115.56 ± 18.12^ab^
	10		14.38 ± 0.62^a^	118.44 ± 20.14^ab^

Note

Values in brackets are calculated using the relation between the standard curves with gallic acid or EC. Mean values in the same column and for each method following by different letters are significantly different (*p* <.05).

Abbreviation: MJ, plain melon juice; ECSMJ, plain melon juice supplemented with epicatechin.

1 ABTS, DPPH and FRAP is expressed as µmol Trolox 100 ml^‐1^.

^2^ Total phenol contents are expressed as mg gallic acid equivalents (GAE) 100 ml^‐1^ by Fast Blue BB (FBBB) and Folin‐Ciocalteau (FC) methods.

In addition, values for EC and ECSMJ antioxidant activity were different for the three methods, but similar for the two samples in each method. Also, these values were approximately 120 (ABTS), 100 (DPPH), and 30 times higher (FRAP) than those of MJ. EC is a flavanol that exhibits the highest radical scavenging activity, significantly more than other flavonoids, as previously reported by Cai *et al*.(Cai et al., 2006).

TPC was determined in MJ and ECSMJ during the storage refrigeration period by the FBBB and FC methods (Table 5). In MJ, the average values were 6 or 14 mg of GAE 100 ml^‐1^, with FBBB or FC, resulting in a quotient value of 0.4. Previously, we determined a quotient value of 2.4,(Rúa et al., 2018) which could be due to the fact that a different batch of melons were used. In this sense, it has been described that although fruits and vegetables are recognized as the best source of the antioxidant diet, the amount and type of each is influenced by a number of factors, including genotype, ontogeny, environment, and postharvest hand (for review refer Salandanan *et al*.).(Salandanan et al., 2009).

The high TPC in ECSMJ, with mean values of 2,184 or 123 mg of GAE 100 ml^‐1^, according to the method used, FBBB or FC, respectively, is noteworthy. The value obtained by the FBBB method of 2,184 mg GA 100 ml^‐1^ is much higher than the added amount of EC (250 mg 100 ml^‐1^), which seems to indicate that the TPC is being overvalued by this method. To check this interference, a test was performed simultaneously with GA and EC, obtaining a linear relationship in both cases with a value of the equation of *y* = 3.62 × 10^–3^ × + 0.29 with GA and *y* = 2.85 × 10^–2^ × + 8.17 × 10^–2^ with EC, the value of the slope ratio being approximately 8. Therefore, this possible interference was avoided by correcting the value of TPC in ECSMJ giving an average value of 273 mg EC 100 ml^‐1^, which is similar to the one added (refer values in brackets in Table 5).

### 3.4 Growth and survival of inoculated bacteria in refrigerated melon juice

**FIGURE 1:**
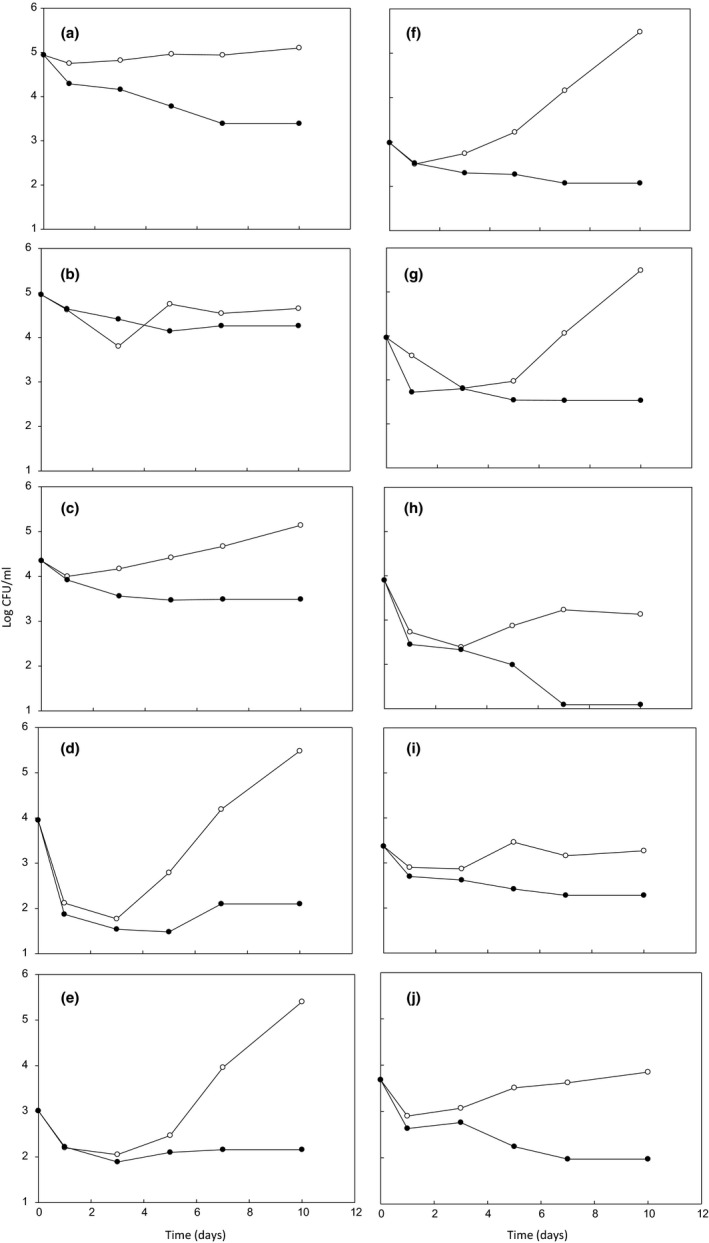
Growth and survival of ten bacterial strains in inoculated MJ (○) and in ECSMJ (●) at 4ºC for 10 days: A, *Lb. rhamnosus* ATCC 53,103, B, *Lc. lactis* 660, C, *En. faecalis* ATCC 29,212, D, *B. cereus* GTE, E, *B. cereus* HRM‐1, F, *L. monocytogenes* CECT 4,032, G, *L. monocytogenes* L 74, H, *E. coli* ATCC 700,728, I, *E. coli* 172, and J, *E. coli* ATCC 25,922

Figure 1 shows the growth and survival of 10 bacteria strains assayed in this study in inoculated MJ and in ECSMJ samples during storage at 4ºC for 10 days. In MJ, the bacteria tested either grew or remained viable; so growth of the two strains of *B. cereus* and of the two of *L. monocytogenes* was observed, after a period of adaptation, with an increase of 1.5 or 2.5 logarithmic units after 10 days of storage. Minor growth was observed for *En. faecalis* (an increase of 0.8 log CFU/mL after 10 days’ storage). No growth was detected in the two PT‐LABs and three *E. coli*, although a proportion of cells remained viable. No growth was observed in ECSMJ for the 10 bacteria in general, but a decrease in the log CFU/mL between 1.1 and 2.8 for the three strains of *E. coli* and between 0.7 and 1.8 for the rest of the strains at 10 days of storage to refrigeration was observed.

It is important to note that pathogenic bacteria presented a decrease in counts during storage in ECSMJ samples compared to MJ, resulting between 1 and 2 log CFU/mL lower for the two pathogenic *E. coli*, and between 3 and 3.5 log CFU/mL lower for the *B. cereus* and *L. monocytogenes* strains after 10‐day storage in comparison with the inoculated MJ. Regarding the two PT‐LAB strains, it was observed that EC produced decreases of 0.4 log CFU/mL for *Lc. lactis* 660 and 1.7 log CFU/mL for *Lb. rhamnosus* ATCC 53,103 when compared with inoculated MJ.

The low resistence of *E. coli* strains to EC, which could be explained by the presence of certain proteins in the external membrane, creates channels for the penetration of low molecular weight compounds.( Cava‐Roda et al., 2012) In particular, *E. coli* ATCC 700,728 (O157:H7) was the most susceptible to EC (no viable cells detected at 10‐day storage), therefore it would be of interest to remove the possible presence of this pathogenic strain in MJ.

We calculated the highest growth potential (δ) in MJ and ECSMJ samples for each bacterial strain during storage at 4ºC for 10 days. MJ was a suitable growth medium (δ > 0.5 log CFU/mL) for six out of the ten bacteria tested: two LAB (*Lb. rhamnosus* ATCC 53,103 and *En. faecalis* ATCC 29,212), two *B. cereus,* and two *L. monocytogenes* strains, which showed δ values between 0.68 and 2.50 after 10‐day storage. However for the rest (one PT‐LAB bacteria and three *E. coli* strains), MJ was not a suitable medium for growth (δ between 0.04 and −1.62). Growth of *L. monocytogenes* on cut cantaloupe or cantaloupe pulp has also been demostrated.(Ziegler et al., 2018) In ECSMJ, the values of δ ranged from −0.7 to −2.8; so the presence of EC counteracted the capacity of MJ as a growth medium for four pathogenic bacteria (the two *B. cereus* and the two *L. monocytogenes*) and *En. faecalis* throughout refrigerated storage.

## 4 CONCLUSIONS

The addition of EC to “Piel de Sapo” MJ provides it with antimicrobial properties that make it a safe food during storage at 4ºC for 10 days. In general, this phenolic compound produces a decrease in counts (log CFU/mL) of the seven pathogenic bacteria during storage, reaching undetectable values for *E. coli* O157: H7 after seven days of storage at 4ºC, while for both PT‐LABs the addition of EC does not prevent their survival during storage. The addition of EC to MJ resulted in a final log concentration of between 4.3 and 2.0 CFU/mL, hence growth was prevented but its effect was largely bacteriostatic, except for *E. coli* O157:H7, which is bactericidal. Also the addition of EC greatly increases antioxidant activity and the low phenolic functional content of this juice, which could have a potential application in its presser vation, also increases the nutritional value of the product.

## CONFLICT OF INTEREST

The authors declare no conflict of interest.

## ETHICAL APPROVAL

Ethics approval was not required for this research.
